# Reading between the Lines: Process Mining on OPC UA Network Data

**DOI:** 10.3390/s24144497

**Published:** 2024-07-11

**Authors:** Markus Hornsteiner, Philip Empl, Timo Bunghardt, Stefan Schönig

**Affiliations:** Faculty of Informatics and Data Science, University of Regensburg, 93053 Regensburg, Germany; markus.hornsteiner@ur.de (M.H.); philip.empl@ur.de (P.E.); timo-bunghardt@t-online.de (T.B.)

**Keywords:** process mining, industrial IoT, business process management, industry 4.0

## Abstract

The introduction of the Industrial Internet of Things (IIoT) has led to major changes in the industry. Thanks to machine data, business process management methods and techniques could also be applied to them. However, one data source has so far remained untouched: The network data of the machines. In the business environment, process mining, for example, has already been carried out based on network data, but the IIoT, with its particular protocols such as OPC UA, has yet to be investigated. With the help of design science research and on the shoulders of CRISP-DM, we first develop a framework for process mining in the IIoT in this paper. We then apply the framework to real-world IIoT network traffic data and evaluate the outcome and performance of our approach in detail. We find tremendous potential in network traffic data but also limitations. Among other things, due to the dependence on process experts and the existence of case IDs.

## 1. Introduction

Industrial Internet of Things (IIoT) technologies have ushered in a new era of manufacturing and industrial processes, offering unprecedented levels of connectivity, automation, and data-driven decision-making. In the heart of these dynamic ecosystems lies the seamless exchange of information among interconnected devices, sensors, and control systems [1]. This intricate web of interactions, facilitated by standard industrial communication protocols such as OPC UA (Open Platform Communications Unified Architecture) [2] and MQTT (Message Queue Telemetry Transport), generates vast volumes of network data, which, until recently, remained an untapped resource for unraveling the underlying operational intricacies [3,4].

In this paper, we delve into the realm of process mining as a transformative approach to extract invaluable insights from collected network data in IIoT environments. Process mining, a field at the confluence of data science, machine learning, and process management, refers to the automated discovery, monitoring, and improvement of process models from event data of IT systems [5]. Event data are used in the research area of process mining to generate and compare process models automatically with the help of process mining algorithms. Event information can be generated by classical IT systems as well as by employees using smart devices, (production) machines, and sensors [6,7,8]. IT systems within an organization create, for example, records of activities performed, messages sent, or transaction data. These event data are compiled into event logs and form the starting point for process mining algorithms.

Using network data to discover business processes is an emerging research area that has recently garnered significant attention [9,10,11,12]. Integrating process mining with network data in IIoT environments has the potential to revolutionize industrial operations by providing a data-driven perspective for optimizing processes, enhancing decision-making, and unlocking the full potential of IIoT technologies [13]. This paper thoroughly investigates this innovative intersection, exploring its theoretical foundations, practical implementation, and transformative impact on industrial operations. It addresses the following research question: “How to mine operational processes from OPC UA network traffic data?” To the best of our knowledge, our approach is the first to focus on rule-based process mining using real-world OPC UA network data. We outline the essential steps to transform unstructured and raw network data into an event log suitable for process mining, with a particular emphasis on collecting, preprocessing, and analyzing network data from IIoT environments. This involves addressing the challenges and complexities associated with handling large-scale, heterogeneous data sources. Through a real-world use case, we demonstrate the practical application of our approach, showcasing how it can generate actionable insights that lead to significant operational improvements. In summary, our contributions are as follows:

- We introduce a novel approach to generate event logs from OPC UA packets for use in process mining.
- We implement a proof-of-concept based on our approach, demonstrating the performance and quality of the process models derived from the generated event logs.
- To the best of our knowledge, we are the first to apply process mining on real-world network traffic data, rather than simulated data, illustrating how this approach can produce actionable insights that translate into operational benefits.

The paper is structured as follows: in Section 2, we present essential basics and related literature on process mining and network traffic data. This is followed in Section 3 by our method to discover business processes in the IIoT. In Section 4, we present the implementation of the method, which we apply to a real-world use case in Section 5. We evaluate and discuss our method in Section 6 regarding performance and quality and conclude the paper in Section 7.

## 2. Background and Related Work

### 2.1. Process Mining and Network Event Data

Event data, generated during business process execution, include details on activities, their sequence, timestamps, and contextual information. Event data are derived from systems like databases, software applications, or sensors and are the foundation for process mining. By combining data mining, machine learning, and process management techniques, process mining analyzes and visualizes event data to reconstruct and model organizational processes. It identifies inefficiencies, bottlenecks, compliance issues, deviations, and improvement opportunities. Process mining relies on event logs as its core input, forming the basis for analyzing and optimizing processes. Network data are precious for process mining due to various reasons:

- Rich Information Source: Network data contain information generated by interconnected devices and systems. They capture interactions and communications between entities, providing a detailed record of activities and their sequence.
- Granularity and Detail: Network data often offer granular insights into the flow of activities and dependencies among different elements within a system. This information can be valuable for reconstructing processes accurately.
- Real-Time and Continuous Data: Networks generate real-time data as activities occur, offering a current and comprehensive view of ongoing processes. This real-time feature allows for immediate analysis of deviations or inefficiencies.
- Comprehensive Coverage: Network data often cover various activities, including structured and unstructured data, allowing for a holistic view of processes.
- Interconnection of Systems: In many cases, processes are interconnected across various systems or devices. Analyzing network data helps understand the interactions and dependencies among these systems, offering insights into end-to-end processes.

Network data, though rich, can be complex and varied, requiring specialized expertise for effective preprocessing, analysis, and interpretation in process mining. Regarding the IIoT, OPC UA is a widely used communication protocol in industrial automation, which is discussed in the following.

### 2.2. OPC UA Protocol

OPC UA (Open Platform Communications Unified Architecture) is a machine-to-machine communication protocol that is widely used in industrial automation systems. It provides a framework for secure and reliable data exchange between various devices and applications in a networked environment. OPC UA supports multiple data encoding formats to represent information during communication. These formats include binary, JSON (JavaScript Object Notation), and XML (eXtensible Markup Language). Each format has its own characteristics and usage scenarios. Binary encoding is preferred for performance-critical applications with limited bandwidth, while JSON and XML are used in web-based and interoperable systems where human readability and compatibility are crucial.

Table 1 provides an overview of the OPC UA packet structure. OPC UA can establish secure channels to ensure data confidentiality and integrity (A). Messages are either encapsulated within this channel or directly transmitted over the network. The message header contains crucial details such as type, size, and encoding (B). The message body holds the actual content, varying by message type (C). OPC UA defines a set of services that allow clients and servers to interact (D). These services are transmitted via the message body and provide functionality for various operations, such as reading and writing data, subscribing to events, browsing the server’s address space, and managing sessions.

**Table 1:** OPC UA Packet Structure.

Secure Channel Layer (A)	Optional
Message Header (C)	fixed size
Message Type	4 bytes
Message Size	4 bytes
Secure Channel ID	4 bytes
Security Flag	4 bytes
Additional Header	variable size
Message Body (D)	variable size
ReadRequest/ReadResponse (E)	variable size

Every OPC UA handshake follows the request and response pattern, as shown by the read operation in Table 2. Besides the requested (e.g., NodesToRead) or transmitted data (e.g., Results), OPC UA packets carry the request handle located in the header, a unique identifier assigned to a client’s request message when communicating with a server. It correlates a request (see Table 2a) and a response (see Table 2b) within a session. The request handle serves three main purposes. First, it allows the client to match the response received from the server to the original request. Second, OPC UA supports asynchronous communication, where a client can send multiple requests to a server without waiting for responses. Third, in case of errors or exceptions during processing, the server includes the request handle in the error response.

**Table 2:** Request and Response Headers. (**a**) OPC UA Read Request; (**b**) OPC UA Read Response.

(**a**)	
Request Header	
Type ID	4 bytes
Request Handle	4 bytes
Timestamp	8 bytes
NodesToRead	variable
(**b**)	
Response Header	
Type ID	4 bytes
Request Handle	4 bytes
Timestamp	8 bytes
Results	variable

### 2.3. Related Work

Process mining is an analytical discipline that aims to discover, monitor and improve real-world processes by extracting knowledge from event logs available in today’s information systems [14]. It bridges the gap between data-centric analysis techniques such as machine learning, data mining and business process management. By leveraging event logs, process mining provides insights into actual process execution and enables organizations to improve efficiency, compliance and overall process performance [5]. One focus within this discipline is the identification and connection of new data sources for process mining as well as the processing and correlation of analyzed unstructured data and events. The aim is to exploit previously untapped potential. Examples of this include text data [15,16], time series [17], sensor data [18,19], or video data [20,21], or, as in this paper, network data. Network data for process mining is a burgeoning research area gaining significant attention (Table 3). Existing studies predominantly use simulated network data from tools like Wireshark (https://www.wireshark.org/ (acccessed on 10 June 2024)) and can be categorized into two event log generation techniques: rule-based and model-based. We explore related works organized by their event log generation techniques in the following.

**Table 3:** Related works on network data-based process mining.

Reference	Input Data	Log Generation	Automation	Model	IIoT
Wakup & Desel [11]	Simulated	Rule-based	◑	Petri net	
Engelberg et al. [9]	Simulated	Rule-based	◑	BPMN	
Hadad et al. [10]	Simulated	Model-based	◑	Event log	
Apolinário et al. [22]	Simulated	Model/rule-based	⬤	BPMN	
Lange & Möller [23]	Simulated	Model-based	⬤	BPMN	
Lange et al. [24]	Simulated	Model-based	⬤	BPMN	
Empl et al. [25]	Simulated	Rule-based	◑	Petri net	✓
Our paper	Real world	Rule-based	◑	BPMN	✓

◑ semi-automated; ⬤ fully automated.

Rule-based Techniques. Rule-based techniques transform captured network traffic into structured event logs through predefined rules, necessitating manual rule definition beforehand. For instance, Wakup and Desel [11] employ filter TCP dumps with predefined rules and use TCPLog2Eventlog for extraction. Engelberg et al. [9] focus on HR recruitment, applying the heuristic miner to capture network data for business processes. Apolinário et al. [22] introduce FingerCI, combining techniques for ICS model construction.

Model-based Techniques. Model-based techniques generate event logs or process models from network traffic data, requiring no human intervention through unsupervised learning. Hadad et al. [10] propose unsupervised learning for event log generation, addressing challenges in activity recognition from network data. Lange et al. [24] introduce MONA, deriving workflows directly from network data without generating event logs.

In contrast to papers that are not related to IIoT, our work contributes to explainable rule-based event log generation and process discovery, focusing on real-world OPC UA network data captured from a manufacturing company’s end-of-line business process. Unlike Empl et al. [25], we investigate the OPC UA protocol instead of MQTT, utilize real-world network data, and do not alter the network traffic, as their approach requires a pre-defined trace identifier. Additionally, we generate event logs without isolating processes and derive processes without relying on inexplicable machine-learning techniques.

## 3. OPC UA Process Discovery Method

**Figure 1:**
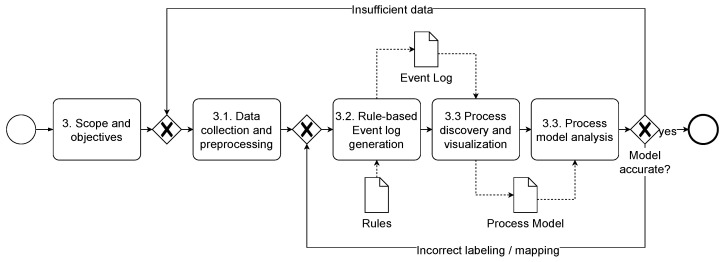
Generic process discovery approach in the IIoT.

To address the lack of a structured approach for process discovery from OPC UA network data, we develop this method in this paper. Process discovery involves obtaining data from running processes, generating event logs, and mining processes from these logs [26]. The challenge lies in abstracting multiple low-level network events into a high-level event log [27]. Following the design science research approach by Hevner et al. [28], we develop an IIoT-specific artifact with CRISP-DM (Cross Industry Standard Process for Data Mining) [29], as illustrated in Figure 1. Further details on the individual phases follow.

Scope. Before starting, it is crucial to determine the scope: the target systems or components (which?), the technique, frequency, and timing (how?), the stakeholders (who?), and the desired outcome of the discovery (what for?). In addition, metrics must be defined to measure whether the scope has been achieved, e.g., which data should be used for the event log, or is there a process model to be compared against the output? The stakeholders involved should document and agree on these metrics to ensure the success [30].

### 3.1. Data Collection and Pre-Processing

Collect. Once the scope and objectives have been defined, we recommend using a passive data collection technique (network sniffing) instead of an active one, as it does not affect the operational processes and aligns with IIoT’s high availability requirements. Passive recording is feasible using appropriate hardware (e.g., a switch with port mirroring) and software (e.g., Wireshark). Regardless of the hardware and software in use, the collected data’s quality (e.g., completeness or encryption) is crucial. Competing with the large data volume, filtering rules (e.g., on ports) ensure alignment with the predefined scope, but when recording an initial snapshot, a full capture is recommended, pushing the understanding of the network further. Last, as the PCAP format might be difficult to handle, it can be transformed into human-readable formats (e.g., XML or JSON).

Understand. Before pre-processing the data, the data analyst must understand the data’s context, e.g., by collecting additional information, such as existing process models, descriptions, expert interviews, asset inventories, or site visits. Afterward, it is crucial to understand the collected data [29]. In IIoT, this means gaining insights into the network topology, IP addresses, ports or protocols. After resolving duplicates, the data analyst can dive deep into the structures of the packets to identify data of interest, such as the case ID for subsequent event logs. The visualization of information (e.g., social network diagram) can also be beneficial before data pre-processing.

Pre-processing. Network data are selected based on scope and objectives. Iteratively approaching the scope and objectives will lead to the desired outcome. Data analysts can assess the model’s quality at each iteration by filtering less data and iteratively refining the selected data for the event log. Event log generation may involve aggregating multiple packets to form activities, especially in client-server architectures. In OPC UA, requests and responses can be matched using the so-called requestHandle (see Algorithm 1). The algorithm generates activities from low-level request-response events. Enriching activity names with human-readable labels ensures understandable process models. For example, if information on the function of a machine is available, replace the IP address and port with this information to increase readability.
**Algorithm 1** Activity generation.**Require:** 
opcua_packets**Ensure:** 
matches1:**procedure** match_packets(opcua_packets)2:     *req* ← empty list3:     *res* ← empty list4:     **for all** *packet* in *opcua_packets* **do**5:          *ip* ← packet.ipdst6:          *port* ← packet.portdst7:          *time* ← packet.time8:          **if** *packet.ttype* == “MSG” **then**9:               find_connection_type(obj)10:              **if** header **then**11:                   *nodes* ← get_node_strings12:                   **if** “RequestHeader” **then**13:                        *req.app*(*time,ip,port,nodes*)14:                   **else**15:                        *res.app*(*time,ip,port,nodes*)16:                   **end if**17:             **end if**18:          **end if**19:     **end for**20:     *matches* ← match_requests(req, res)21:     **return** sort_by_time(matches)22:**end procedure**

### 3.2. Rule-Based Event Log Generation

After generating activities from filtered, aggregated, and labeled network packets, the next step is identifying each activity’s process instance and generating an event log. Mandatory information of an event log includes the (1) case ID, (2) timestamp, and (3) activity name. The case ID is a unique identifier that identifies a process instance or a run and is assigned to all activities involved. Timestamps indicate the event’s occurrence and provide information on sequential or parallel activities. While an event can have different activity names, non-uniqueness within the same process run is permitted. Optional information complements an event log, including information about the resource, e.g., the name of the actuator executing an activity. In the IIoT, we find physical processes and machines handing over products. We can refer to each product traveling through this process as a process instance, while a new process instance is created when it first appears in the network traffic. Each product has a unique identifier, ideal as a case ID. As not every packet carries the product identifier, pseudocode in Algorithm 2 details the event log generation based on the case ID assignment. Automatically assigning activities ensures consistency over the process and the event logs. Experimenting with different case IDs (in the case of appropriate candidates) further allows the comparison throughout the event logs.
**Algorithm 2** Event log generation.**Require:** 
matches**Ensure:** 
cases1:**procedure** add_case_id(matches)2:      *prod_id* ← ITEM_ID3:      *ip_to_case_id* ← empty dictionary4:      *cases* ← empty list5:      **for all** *match* in *matches* **do**6:            *time* ← *match.time*7:            *ip* ← *match.ip*8:            *port* ← *match.port*9:            *nodes* ← *match.nodes*10:           *case_id* ← *None*11:           **for all** *node* in *nodes* **do**12:               **if** prod_id in *node.keys()* **then**13:                    *case_id* ← *node(prod_id)*14:                    *ip_to_case_id[ip]* ← *case_id*15:                    **break**16:               **end if**17:           **end for**18:           *case_id* ← *ip_to_case_id.get(ip)*19:           cases.app(time, ip, port, case_id)20:      **end for**21:      **return** *cases*22:**end procedure**

### 3.3. Process Discovery, Visualization and Analysis

The derived event log is the basis for applying process mining techniques and enables identifying and visualizing processes and process instances. For example, process mining discovery techniques include heuristic, alpha, and inductive miners, which produce different outcomes (e.g., BPMN or Petri net). Each outcome, when visualized, shows different process perspectives. A direct follows graph creates an overview of process instances and dimensions (e.g., frequency or performance). The BPMN notation (and notably extended options with context-specific variables) focuses more on business processes [31].

Data analysts can interpret the results regardless of the notation or process mining technique used. This way, deviations between the discovered and target processes can be identified, e.g., bottlenecks. Visualizations also help to uncover optimization potential. For informed decision-making, stakeholders can enrich the process models with expert knowledge if required. An inaccurate model (e.g., inadequate data or pre-processing) may result in returning to an earlier phase.

## 4. OPC UA Mining Implementation

This section introduces the Python implementation details of the event log generation using the OpcuaPacketAnalyzer class. This analyzes OPC UA network packets, extracting relevant information and generating event logs. The implementation is available on GitHub (https://github.com/philipempl/opcua-mining). It loads OPC UA data from a JSON file, extracts data from packets at various ISO/OSI layers, matches request/response handles in OPC UA packets, and generates CSV event logs. These event logs serve as the foundation for subsequent analysis.

### 4.1. Software Design

**Figure 2:**
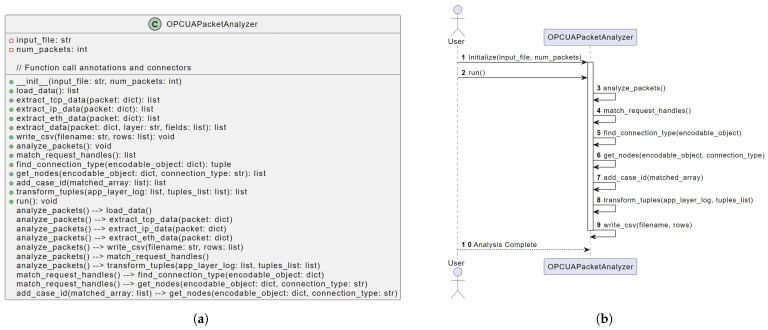
Implementation design of the OpcuaPacketAnalyzer class. (**a**) Class diagram. (**b**) Sequence diagram.

In Figure 2, we present a visual representation of the OpcuaPacketAnalyzer class structure and relationships (see Figure 2a). In the class diagram, we can derive the structure of the OpcuaPacketAnalyzer class, including its attributes and methods. The relationships between methods are depicted to provide a high-level overview of how they interact. A sequence diagram depicts the interactions and flow of control between objects and actors. In our case, we use a sequence diagram to illustrate how the OpcuaPacketAnalyzer class is invoked and how its methods interact (see Figure 2b). The sequence diagram shows the actions when users interact with the OpcuaPacketAnalyzer. The user initializes the class, runs the analysis, and triggers various internal methods to perform specific tasks, which we detail in the following.

### 4.2. Implementation Details

We provide detailed explanations of key methods and functionalities of the OpcuaPacketAnalyzer class in the following:

Entrypoint. The analyze_packets() method is the entry point for event log generation, orchestrating data extraction, request handle matching, case ID assignment, and event log generation. It structures OPC UA packets for process mining and analysis.
Data Loading. The load_data() method loads OPC UA communication data from a Wireshark JSON file, ensuring availability for subsequent methods.
Data Extraction. Utilizing extract_tcp_data(), extract_ip_data(), and extract_eth_data(), this step extracts relevant data from packets at various ISO/OSI layers.
Request Handle Matching. The match_request_handles() method matches request handles in OPC UA packets, establishing relationships between requests and responses and creating activities.
Event Log Generation. The write_csv() method generates CSV event logs from extracted data for process mining or visualization.
Case ID Assignment. The add_case_id() method assigns case IDs to matched arrays of OPC UA packets based on keys, facilitating subsequent process mining techniques.

## 5. Use Case: End-of-Line Process

In this section, the methods from Section 3 to a real industrial use case are applied, demonstrating their application and relevance to OPC UA network data. We use the OpcuaPacketAnalyzer class in a scenario involving an automotive supplier’s end-of-line process, which includes robotic inspections, laser engraving, and cleaning. We examine each method phase and discuss appropriate measures. The dataset includes activities from four machines and a central process control system, providing a real-time process snapshot. The dataset comprises a total of 33 process instances, 30 of which are completed. A completed process instance signifies that the production of a part has commenced and reached a definitive conclusion. This conclusion can either indicate the completion of the entire process, resulting in a finished part or the termination of the process at an intermediate stage due to quality defects or other issues, leading to the ejection of the part. Our goal is to investigate the feasibility of mining this IIoT business process from OPC UA data.

### 5.1. Data Collection and Pre-Processing

Collect. We collect the data in real-time using a Raspberry Pi (https://www.onlogic.com/eu-en/computers/industrial/fanless/factor-200/) connected to the switch responsible for network communication. Using port mirroring, the Raspberry Pi captures and stores 30 minutes of network traffic in plain text on a USB hard disk. This created a snapshot of the network communication during live operation in PCAP format.

**Figure 3:**
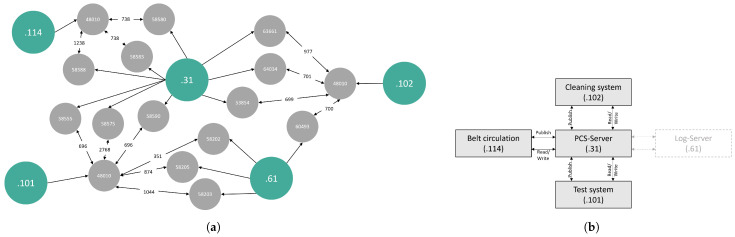
Representation of visualizations. (**a**) Network communication frequency based on IP addresses (green) and ports (grey). (**b**) Human-readable machine labeling of IP addresses.

Understand. Initially, we attempted to read the PCAP file using pyshark (https://github.com/KimiNewt/pyshark), but faced limitations, such as no support for OPC UA. We then exported the network data with Wireshark to JSON, specifying relevant OPC UA service ports. The Wireshark OPC UA extension aids packet interpretation, enabling the creation of the network structure (see Figure 3a) for an overview. We identified the central network’s IP address as .31 for the Process Control System (PCS) server. Among 24,445 OPC UA packets, we found 24,421 OPC UA message packets and 24 OpenSecureChannelRequest packets, which we did not further analyze. In total, 9244 packets have been sent by the PCS, the PCS has received 9247 packets, and 2965 were sent to the protocol server. The network data reveal that the PCS requests machine information through read and write requests. Publish and response packets lack production-relevant content, possibly due to an OPC PubSub-based notification system. Publish request packets originate from the PCS or the log system and are addressed to the cleaning, conveyor, and test systems, resulting in publish response packets. As the packet timestamps lack unique polling information, we exclude publish and subscribe packets from the event generation process.

Pre-processing. Following the contextual analysis of the packets, we initiate pre-processing. First, we exclude packets containing the protocol server and focus on OPC UA packets between machines and the PCS. We apply the request handle matching algorithm to create activities by aggregating OPC UA packets with matching request handle. For better human readability, we assign labels using IP addresses with device type mapping (IP address:label). In Figure 3b, these labels, like .31:PCS server, serve as activity names in event logs, enhancing readability.

### 5.2. Rule-Based Event Log Generation and Process Mining

Next, we generate the event logs for the use case. We identify a product identifier (CanProduce.ITEM_ID) in the network traffic and use it as the case ID for the event log generation algorithm. The choice of case ID depends on the use case, which emphasizes the need to understand the data. The event log is then written to CSV files. After creating the event log, we apply process mining techniques. Appendix A shows the directly-follows graph of the event log. Since the alpha, heuristic and inductive miner use different algorithms, their results vary. Each process model has been evaluated for accuracy by process experts, with the result that all reflect reality to some degree. However, the process experts encounter difficulties when evaluating low-level network events.

## 6. Evaluation

As already shown that mining processes from OPC UA network data are feasible, we aim to assess the scalability and quality of our approach. To assess mining capabilities and model quality in the OPC UA context, we implement experiments within a Jupyter notebook, available on GitHub (https://github.com/philipempl/opcua-mining). Using a MacBook Pro 2021 with an Apple M1 Pro chip, 8 cores, and 16 GB of memory, we employ experiments on the OPCUAPacketAnalyzer class, analyzing OPC UA packets to generate event logs. This class extracts data from different ISO/OSI stack layers, generating logs for process mining algorithms. Performance evaluation involves generating event logs of varying sizes to understand scalability. Quality metrics such as replay fitness, precision, generalization, and simplicity gauge model performance. Collaboration with a process expert validates real-world accuracy and relevance, enriching results and fortifying practical implications.

### 6.1. Results

**Figure 4:**
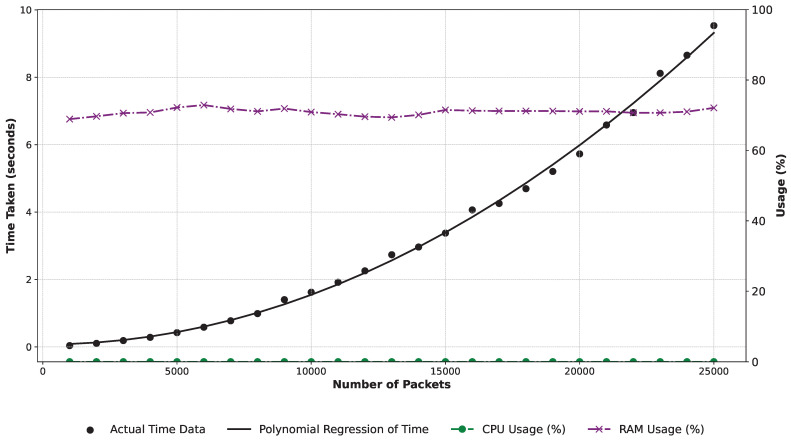
Performance analysis: time taken for analysis, CPU and RAM usage.

Event Log Generation Performance. Our experimental setup explores OPC UA packet processing performance by incrementally analyzing varying loads. Key metrics include time, CPU, and RAM usage. We start with 1000 packets, increasing by 1000 in each run until dataset exhaustion. Visualizing the results in Figure 4, packet analysis time shows a quadratic relationship with packet count, confirmed by a polynomial regression (black line). As expected, processing time increases with more packets. CPU and RAM usage (green and magenta lines) remain consistent, with occasional RAM spikes and steady CPU usage. Results indicate a significant computational demand increase with rising packet count. The polynomial regression in the experimental setup is as follows:

$$T\left(p\right)=1.4840\times 10^{-8}p^{2}-1.1514\times 10^{-6}p+0.0728$$

The polynomial regression trendline offers a predictive insight, where T(p) is the time taken, and *p* is the number of packets, suggesting that for larger data sets, resource allocation should be planned judiciously to ensure optimal performance. For instance, generating an event log for 1,000,000 OPC UA packets requires approximately four hours, which is appropriate as it is the initial step towards process mining and deriving process models, which is relatively fast. The observed CPU/RAM usage trends further emphasize the importance of efficient resource management, when dealing with substantial packet loads.

**Figure 5:**
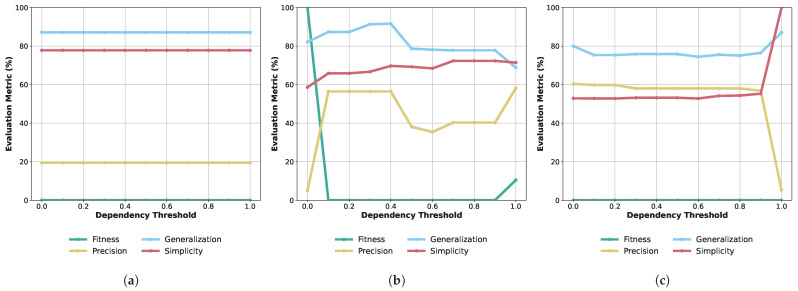
Process discovery algorithms’ quality with varying thresholds. (**a**) Alpha miner; (**b**) Heuristic miner; (**c**) Inductive miner.

Process Model Quality. Within our setting, we compare the quality of three process discovery algorithms, the Alpha miner, Heuristic miner, and Inductive miner, across varying dependency thresholds on our OPC UA data (see Figure 5). Therefore, we use established quality metrics: replay fitness, precision, generalization, and simplicity. Replay fitness measures how accurately the discovered model can reproduce the event log. Precision indicates how well the model represents the event log. Generalization measures how well the discovered model can handle variations and unseen instances beyond the event log. Simplicity quantifies the level of complexity required by the model to represent the event log. In the following, we discuss those metrics.

This threshold ranges from 0 to 1 and represents the minimum required dependency between activities to establish a causal relationship. Note, that the Alpha miner does not rely on dependency thresholds, resulting in horizontal lines. For the *Alpha miner* (Figure 5a), it consistently shows 0% fitness, indicating poor alignment with the event log. Precision, generalization, and simplicity metrics remain stable but at low values ( 19.4%, 87.1%, and 77.8%, respectively). This consistency indicates its limited adaptability. The *Heuristic miner* (Figure 5b) exhibits varied performance. At a threshold of 0, it achieves 100% fitness, declining sharply at higher thresholds. Precision peaks at 56.4%, with an upward trend in generalization. Simplicity fluctuates but remains within the mid-60% to mid-70% range. The *Inductive miner* (Figure 5c) shows intriguing results. At lower thresholds, it has 0% fitness, comparable to the Alpha miner. Precision starts at 60.3% and declines with higher thresholds. Generalization and simplicity fluctuate but within a tight range. In summary, the Heuristic Miner is highly adaptable but the Inductive Miner offers a balanced performance in precision, generalization, and simplicity. The Alpha miner, while stable, lacks alignment with the log. Considering these nuances is vital for selecting an optimal miner in practical applications.

Operational insights. We also gain insights from the continuous evaluation of processes through our industrial collaboration. An initial statement from the process expert is that “he would never have believed that we could get so close to the real process using only network data”, which led to an internal rethink about the importance of network data for operational benefits. In addition, within the analysis of the network data, we identified further potential for process optimization. For example, in addition to OPC UA, we discovered that a server regularly searches for printers in the network, which reduced the performance of the network. There were also indications of typing errors in naming and variations in variables, which were identified.

### 6.2. Discussion

Limitations. While our research highlights the benefits of process mining in OPC UA network data, we acknowledge limitations that may impact the generalizability of our findings. First, our paper assumes the availability of network data in plain text. Encryption is sometimes used in real-life scenarios, which could conceal important information. Secondly, the method relies on a product ID for tracing and differentiating process instances. In cases of absent or inconsistent identifiers, mined process accuracy and completeness may be limited. Lastly, our dataset, covering only 32 unique process instances, may not represent the diversity of processes in more complex industrial settings, affecting the robustness and applicability of our insights.

Scientific Impact. In the evolving realm of process mining, our paper marks a paradigm shift, breaking away from conventional approaches. We pioneer the application of process mining to OPC UA data, showcasing its feasibility and effectiveness while highlighting key challenges, notably in data availability. This revelation emphasizes that datasets suitable for process mining are more extensive than previously believed. Our findings have broad applicability, such as in cybersecurity, where process models can enhance network intrusion detection or ensure compliance [31,32]. Last, our insights into OPC UA processes offer valuable nuances for future benchmarking studies.

Practical Impact. In the field of process mining, the decoding of OPC UA network data holds transformative potential for gaining insights into operational processes. Although our models currently have qualitative limitations, they already reflect real process behavior at the end of the production line. A larger volume of data would enable more meaningful models. While process experts are able to develop an understanding of the macro level, they may lack the granularity of network-level events. Accurately identifying process starting points is critical to aligning the mapped processes with the experts’ understanding. Our 30-min capture shows that a one-week snapshot can reveal essential details for in-depth analysis. By bridging the gap between high-level process knowledge and complex network traffic patterns, organizations can realize the full potential of process mining.

In order to assess the accuracy of our process mining approach, we also collected the process manually by applying the methods of document analysis, interview and observation. To conduct this, we first examined two hours of available documents, then conducted a total of three interviews with two process experts over a total period of three hours and then observed the process on-site for two hours. We found that there was only a small difference between the manually recorded process and the real world, although this could be closed by the automatically recorded processes. Overall, however, it can be said that an automated survey has significant advantages over a manual one in terms of effort and the associated costs. An automated recording and subsequent semi-automated investigation requires significantly fewer experts than interviews lasting several hours or an observation. In our opinion, an automated mining procedure and subsequent comparison by means of observation would be the most cost and effort-efficient way to survey processes in the IIoT.

## 7. Conclusions

Our research taps into the rich potential of network data in the IIoT, an area that has not been fully explored for generating event logs and uncovering business processes. To the best of our knowledge, we are the first to introduce a method that reveals IIoT processes based on (OPC UA) network traffic data. Our method not only advances academic research, allowing for more detailed comparisons and improvements (like benchmarking), but it also shows practitioners the real value of network traffic data. We developed an open-source prototype that represents a significant shift in process mining, offering a transparent and understandable way of mining OPC UA network data. Despite facing challenges like network encryption and working with a relatively small dataset, our findings are promising. They reveal that our process models accurately reflect a real-world use case at quite high quality with relatively good performance. In our discussion, we emphasize the importance of using larger datasets for more precise results. We are excited to follow future research in this area, confident that network traffic data are poised to unlock new opportunities in process mining and beyond.

## Figures and Tables

**Figure A1:**
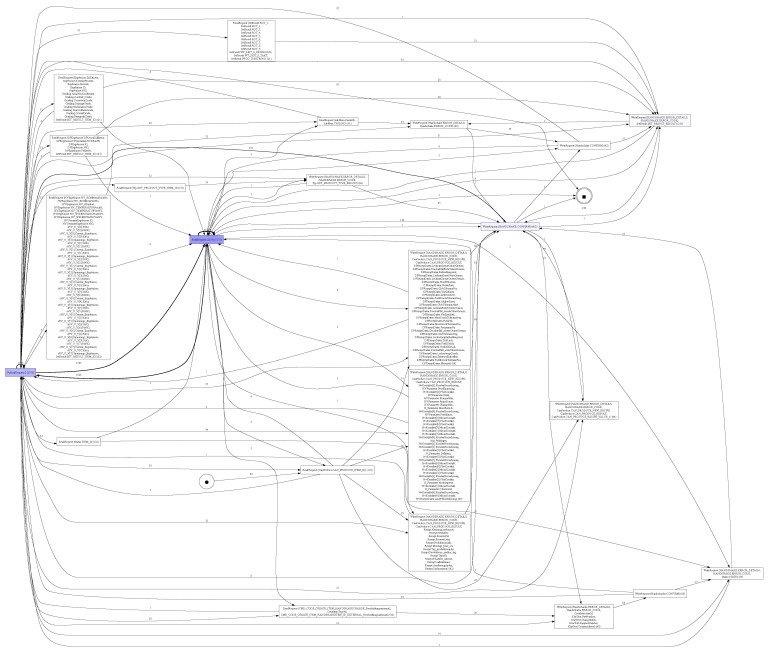
Cluttered directly-follows graph of the end-of-line process.

## Data Availability

The datasets presented in this article are not readily available, as the data used was recorded in the real operations of an industrial company and its cybersecurity policies do not allow the publication of internal information. Nevertheless, all implemented artifacts presented in this paper are available on Github at https://github.com/philipempl/opcua-mining.

## Appendix A. Directly-Follows Graph of the End-of-Line Process

The directly follows graph (DFG) visualizes the sequence and frequency of events or activities in a given process. In this specific DFG, nodes represent distinct activities, such as *PublishRequest* and various *ReadRequest* operations with associated parameters. Directed edges between nodes signify the order in which these activities occur. For instance, an edge from *PublishRequest* to a *ReadRequest* indicates that the *PublishRequest* activity directly precedes the *ReadRequest* activity in the process sequence. Furthermore, numerical annotations on the edges, like 1588 or 1505, represent the sequence frequency, denoting how many times another directly followed one activity in the observed data. The nodes’ parameters detail transferred data within the respective OPC UA tuples. This DFG provides insights into the common paths and patterns of the end-of-line process but is still cluttered.
